# PReS-FINAL-2044: TNF-alpha inhibitors in treatment of children with systemic form of juvenile idiopathic arthritis

**DOI:** 10.1186/1546-0096-11-S2-P57

**Published:** 2013-12-05

**Authors:** ES Zholobova, OU Konopelko, MN Nikolaeva, OS Rozvadovskaya

**Affiliations:** 1 Department Of Pediatric Rheumatology, First Moscow Medical State University I.M. Sechenov, Moscow, Russian Federation

## Introduction

From 2005 to 2011, children with the systemic form of juvenile idiopathic arthritis (JIA), due to the lack of efficacy of standard antirheumatic therapy, received TNF inhibitors, because there were no drugs registered for the treatment of systemic JIA (IL6, IL1 inhibitors) in Russia.

## Objectives

Assessment and comparison of efficacy and safety of TNF-alpha inhibitors (infliximab and etanercept) in the treatment of children with the systemic form of JIA.

## Methods

32 patients with the systemic form of JIA were enrolled in the study. 19 patients received infliximaband 13 received etanercept. All patients had high degree (III) of disease activity. Average disease duration was 7,2 ± 3,3 years. Before administration of TNF-alpha inhibitors, all patients received conventional immunosupressive therapy with 2 or more drugs. In the beginning of the disease, systemic manifestations, such as fever, hepatomegaly, lymphadenopathy, leukocytosis, were observed in 100% of children, rash in 53% in both groups, persistent joint syndrome in the infliximab treatment group was seen in 84% of patients, in the etanercept group - in 73% of children. At the moment of prescription of TNF - alpha inhibitors, the mean number of active joints in the whole group was 20 ± 5, the number of joints with restriction of function - 25 ± 7, ESR - 38 ± 12, C-reactive protein - 6,2 ± 3,4. The drugs were used in standard doses. For assessment of efficacy of performed therapy, "pediatric" criteria of the American College of Rheumatology were used: 30%, 50% and 70% therapy response, that is ACR pedi-30,-50,-70,-90. The criteria ACR pedi were assessed 6 and 12 months after the therapy beginning. Achievement of ACR pedi-30,-50 was regarded as an insufficient response reaction to the therapy being conducted, ACR pedi-70 and higher - as a good response reaction (achievement of medicament remission or low disease activity).

## Results

In the infliximab treatment group, the good response was achieved in 31% of patients by the 12^th ^month of therapy. In children with domination of visceral manifestations in the beginning of the disease, infliximab therapy was ineffective. In the etanercept group, the good response was achieved in 55% of patients by the 12^th ^month of therapy. In three patients, earlier received infliximab therapy without distinct effect, etanercept treatment appeared to be unsuccessful too. The highest effectiveness of the drugs was registered in children without extraarticular manifestations of systemic JIA at the baseline. Further, all children with insufficient response to the therapy with TNF-alpha inhibitors were transferred to tocilizumab.

## Conclusion

Therapy with TNF-alpha inhibitors has appeared to be insufficient in the treatment of the systemic form of JIA. Administration of TNF-alpha inhibitors is justified in children without extraarticular manifestations of systemic JIA at the moment of prescription of the drug. No statistically reliable difference in efficacy has been revealed between infliximab and etanercept groups (p > 0,05). The safety profile of etanercept is significantly higher than that one of infliximab (p ≤ 0.05). The received results reconcile with data earlier published in the scientific literature

## Disclosure of interest

None declared.

